# Parental Feeding Practices, Weight Perception, and Children’s Appetitive Traits Are Associated with Weight Trajectories in Preschoolers: A Longitudinal Study in China

**DOI:** 10.3390/nu16213746

**Published:** 2024-10-31

**Authors:** Yujia Chen, Fangge Qu, Xiaoxue Wei, Xinyi Song, Ruxing Wu, Jian Wang, Yang Cao, Ningyuan Guo, Wenzhe Hua, Xianqing Tang, Daqiao Zhu

**Affiliations:** 1 School of Nursing, Shanghai Jiao Tong University, Shanghai 200025, China; y-yoga@sjtu.edu.cn (Y.C.); qufangge@126.com (F.Q.); weixiaoxue1998@163.com (X.W.); songxinyi@sjtu.edu.cn (X.S.); wu_ruxing@163.com (R.W.); nyguo@shsmu.edu.cn (N.G.); huawenzhe1992@163.com (W.H.); 2 Florence Nightingale Faculty of Nursing, Midwifery and Palliative Care, King’s College London, London SE1 8WA, UK; k21087857@kcl.ac.uk; 3 Clinical Epidemiology and Biostatistics, School of Medical Sciences, Örebro University, 70182 Örebro, Sweden; yang.cao@oru.ce; 4 Department of Children’s Disease Prevention, Jinyang Community Health Service Center, Shanghai 200136, China; tangxianqing@126.com

**Keywords:** preschooler, weight change, group-based trajectory model, feeding practices, appetitive traits

## Abstract

Objective: This study aimed to examine the trajectories of body mass index-for-age z-score (BAZ) in preschoolers and its association with parental feeding practices, weight perception, and children’s appetitive traits. Methods: A total of 433 preschoolers and their parents from eight public kindergartens in Shanghai were assessed annually over two years. A group-based trajectory model was employed to identify distinct BAZ patterns. Logistic regression was utilized to investigate the baseline factors associated with the BAZ trajectories. Results: Three distinct BAZ trajectories were identified among the preschoolers: “low-stable group” (*n* = 154, 37.3%), “moderate-stable group” (*n* = 214, 47.3%), and “progressive overweight and obesity group” (*n* = 65, 15.4%). The children perceived as overweight and obese by parents (OR = 10.57, 95% CI: 4.89–22.86), and those with lower satiety responsiveness at baseline (OR = 0.86, 95%CI: 0.76–0.97) were more likely to fall into the progressive overweight and obesity group as opposed to the moderate-stable group. Conversely, the children perceived as underweight by parents (OR = 457, 95%CI: 2.71–7.70) had a higher likelihood of being in the low-stable group rather than the moderate-stable group. Conclusions: This study unveiled three unique body weight trajectories among preschool children. Parental perception of children’s weight and lower satiety responsiveness were associated with preschoolers’ subsequent weight change, while parental feeding practices were not associated.

## 1. Introduction

Early childhood overweight and obesity (OWOB) has been a growing public health concern in many countries. According to WHO (2023) [1], the global prevalence of childhood obesity has significantly increased and become an epidemic since 2000, with an estimated 5.6% (about 37 million) of children under 5 years old being overweight or obese. Nearly half of these children reside in Asia. In China, the prevalence of OWOB among children under the age of 6 reached 10.4% in 2019 [2]. Children with OWOB are at higher risk of metabolic disorders, cardiovascular abnormalities, and other psychological problems compared to children with a healthy weight [3,4,5]. Therefore, it is critical to identify the risk factors to prevent OWOB among preschool children.

It is widely recognized that children’s body weight is affected by both genetic and environmental factors. As an essential part of the family’s dietary environment, parental feeding practices have been proven to be closely related to children’s weight [6,7,8,9,10]. Parental feeding practices are defined as intentional strategies employed by parents to directly shape their children’s eating behaviors, such as restrictive feeding, pressure to eat, and monitoring [11,12]. A systematic review of 31 studies [8] showed that parental restrictive feeding and pressure to eat were associated with children’s unhealthy weight, and this association was mostly examined in cross-sectional studies (*n* = 23). However, cross-sectional studies cannot provide causal inferences between variables. Longitudinal studies on parental feeding practices and child weight were limited and reported mixed findings. Two longitudinal studies conducted in Spain and Taiwan [7,9] indicated that mothers who monitored their children’s diet reduced the risk of subsequent overweight in school-age children. On the contrary, other studies [13,14] reported no association between parental feeding practices and child weight. These inconsistent results across studies might be due to the difference in the factors included in the model and the heterogeneity of the study sample [11,14].

According to the behavioral susceptibility theory [15,16] and Russell’s biopsychosocial model of the development of eating behavior and weight in childhood [17], we hypothesized that appetitive traits could mediate the pathways between genetics, environment, and weight gain. Food responsiveness (FR, the primary driver of eating onset) and satiety responsiveness (SR, the primary driver of eating offset) are the behavioral manifestations of children’s appetitive traits [18]. Several cross-sectional and longitudinal studies have indicated a positive correlation between FR and children’s weight and a negative correlation between SR and children’s weight [10,18,19]. As a result, it is important to consider children’s appetitive traits when investigating the association between parental feeding practices and OWOB.

The existing studies on parental feeding practices, children’s appetitive traits, and children’s weight were almost always conducted in Western countries [6,20], though parenting beliefs and feeding styles are embedded in and influenced by cultural norms. Caregivers’ feeding practices may differ across different countries and cultural contexts. In China, parents often neglect childhood OWOB [21,22], probably due to the traditional notion that increased food intake fosters greater growth. This attitude can result in feeding practices such as overfeeding that might interfere with children’s innate responses to hunger and fullness and affect their weight and overall health.

Consequently, it is necessary to examine how parental feeding behaviors and children’s appetite characteristics influence weight fluctuations in children in the Chinese cultural context.

Our previous cross-sectional study on preschoolers’ mothers [23] indicated that the maternal perception of children’s weight influenced maternal feeding practices. That is, mothers who perceived their children as overweight or obese tended to restrict their children’s diets and were less inclined to pressure them to eat. The current study was built on our previous work to investigate the role of the parental perception of children’s weight on weight changes in a cohort of preschoolers.

Considering the heterogeneity of body weight change across individuals, this study used group-based trajectory modeling (GBTM) to analyze the distinct trajectories of preschoolers. GBTM differs from the traditional variable-centered analysis, focusing on individual weight changes over the entire period.

Overall, this study aimed to identify the weight trajectory of preschool children and analyze its correlates, particularly the role of parental feeding practices, the parental perception of children’s weight, and the children’s appetitive traits on childhood weight changes.

## 2. Materials and Methods

### 2.1. Study Design and Participants

This longitudinal study tracked weight changes among Chinese preschool children utilizing a convenience sample from eight public kindergartens in Pudong District, Shanghai. All the children from the junior class and their parents were invited to take part at baseline in October 2020 with annual follow-up until kindergarten graduation (junior class T0, middle class T1, and senior class T2). The sample size was determined by the G*Power software version 3.1. setting an α (two-tail) at 0.05 and a power at 0.95. Consequently, the necessary minimum sample size was 224 based on a similar study [24] of school-aged children which examined the association between the maternal perception of children’s weight and children’s weight change, identifying an effect size of 1.76 (95% confidence interval (CI):0.72,1.19).

**Figure 1:**
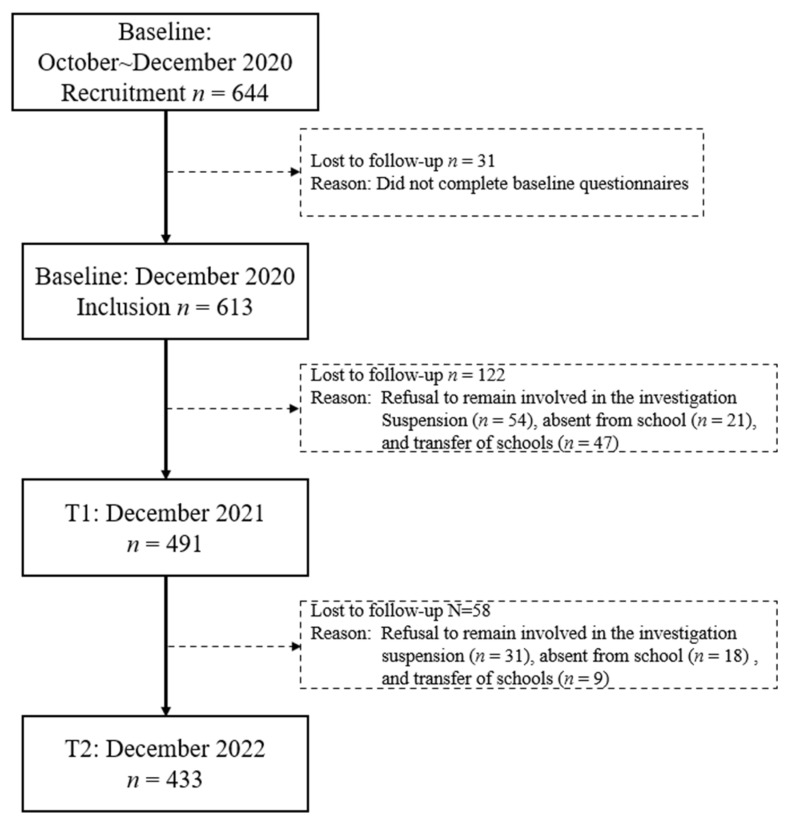
Flow chart of the study participants.

A total of 644 preschool children and their caregivers were initially recruited. In this study, the parent–child relationship specifically referred to parents living with their children for a long time, and parents are the main ones responsible for the children’s diet. The exclusion criteria were as follows: parents who were not the primary caregivers, children with nutrition-related diseases (e.g., fever and diarrhea), and unqualified questionnaires. Overall, 433 (70.64%) preschool children completed the collection of objective data for height and weight at three time points. There were 180 children lost to follow-up (29.36%) due to the following reasons: absence, changing schools, and declining to participate in the follow-up investigation (Figure 1). Ethical approval for this study was obtained from the Ethics Committee of Shanghai Jiao Tong University School of Medicine (registration number: SJUPN-201908).

### 2.2. Procedures

The investigation team consisted of researchers, community pediatricians, nurses, and kindergarten teachers. The height and weight of the children were measured by trained community pediatricians visiting the kindergartens. All the paper questionnaires were distributed and collected in class by trained kindergarten teachers. All the parents provided written informed consent at each assessment point.

### 2.3. Measurements

#### 2.3.1. Children’s Actual Weight

The children’s weight (in kilograms) and height (in centimeters) were measured and collected by trained doctors and nurses in the child health unit of the community hospital. According to the WHO guidelines [25], the children’s BAZ was calculated using the software WHO Anthro (for 2- to 5-year-old children) and Young Growth Curve (for 5- to 6-year-old children). BAZ was categorized into three groups: underweight (z-score < –2), normal weight (–2 ≤ z-score ≤ 1), and overweight or obese (z-score > 1). The BAZ trajectory reflects the potential dynamic changes in weight during children’s growth and development.

#### 2.3.2. Parental Perception of Children’s Weight Status

Parental weight perception was assessed with the question, “How would you describe your child’s weight?” [26]. The parents responded on a 5-point Likert scale, ranging from 1 (very underweight) to 5 (very overweight). The score was re-categorized into the following groups: underweight (≤2), normal weight (=3), and overweight or obese (≥4).

#### 2.3.3. Parental Feeding Practices

Parental feeding practices over the past month were assessed using the Chinese Preschooler’s Caregivers’ Feeding Behavior Scale (CPCFBS) [27] and the Chinese version of the Child Feeding Questionnaire (C-CFQ) [28]. These two scales were used to assess six types of feeding practices: four items of content-restricted feeding (strict limitations on the children’s access to foods or opportunities to consume unhealthy foods), six items of encouraging healthy eating (the extent to create various ways for children to try fresh or healthy foods from meal preparation to the dining process), seven items of behavior-restricted feeding (the impact of parents’ demonstrations on children’s eating content, attitudes, and behaviors), two items of using food as a reward (the use of desired foods as a way to regulate the children’s eating or behaviors), three items of pressure to eat (insists, demands, or physical struggles with children to get the child to eat more food), and four items of monitoring (the degree of supervision over their children’s eating). Each item was worded in terms of the frequency of practice, and response options ranged from 1 (never) to 5 (always). Each subscale score was determined by calculating the average of all the items within that subscale. In this study, Cronbach’s alpha was 0.617–0.872.

#### 2.3.4. Children’s Appetitive Traits

Children’s appetitive traits over the past month were measured using the Chinese Preschoolers’ Eating Behavior Questionnaire (CPEBQ) [29]. CPEBQ assesses two types of appetitive traits: five items of satiety responsiveness (children with small appetites get full quickly) and six items of food responsiveness (children’s tendency to seek food when they see or smell it). Each item was worded in terms of the frequency of practice, and response options ranged from 1 (never) to 5 (always). The score for each subscale was calculated as the mean of all the items included in that subscale. In the current study, Cronbach’s alpha of SR and FR was 0.680 and 0.734, respectively.

#### 2.3.5. Demographic and Family-Related Variables

Demographic and family-related variables were collected using a self-reported questionnaire, including the children’s birth date, sex and parental age, weight, height, education level, family type, number of children, and annual household income. Parental BMI was calculated as weight (in kilograms) divided by the square of height (in meters).

### 2.4. Statistical Analysis

The expectation–maximization algorithm (EM) was used for missing value imputation [30]. All the continuous variables followed a normal distribution. Mean and standard deviation (SD) and count and percentage were used to describe the continuous and categorical variables, respectively. Group-based trajectory modeling (GBTM) was used to identify potential subgroups within a population exhibiting distinct trajectories or change patterns [31]. The best-fit trajectory model was determined according to the following indices: (i) the Bayesian information criteria (BIC) (a smaller absolute value of BIC indicating a better model fit); (ii) fit statistics (the *p*-values of model parameters and the confidence intervals of trajectory estimates); (iii) the average posterior probability (AvePP; above 0.7 indicated optimal fit); (iv) the simplicity of the trajectories (a minimum sample size of at least 5% of each subgroup); (v) the interpretability of the trajectories. After determining the GBTM groups, a one-way analysis of variance or chi-square test was used for group comparisons. A multinomial logistic regression analysis was used to explore the factors associated with the different weight trajectories of preschool children. The associations were quantified using an odds ratio (OR) with a corresponding 95% CI. Data coding, cleaning, and statistical analyses were performed using IBM SPSS Statistics version 26.0 and Traj command [32] in Stata Statistics version 17.0. A two-tailed *p* < 0.05 was regarded as statistically significant.

## 3. Results

### 3.1. Participants Characteristics

A total of 433 children were included in the study, with 232 males (53.6%) and 201 females (46.4%). At baseline, the mean age was 3.70 years (SD = 0.29), and the mean BMI score was 0.13 (SD = 0.88). The majority of the participants were mothers (*n* = 330, 76.2%). Most family types were stem families (living with grandparents, *n* = 356, 82.22%), and over half reported an annual income of more than RMB 300,000 (about USD 41,700) (*n* = 250, 57.73%) (Table 1).

### 3.2. Weight Trajectories of Preschool Children

**Figure 2:**
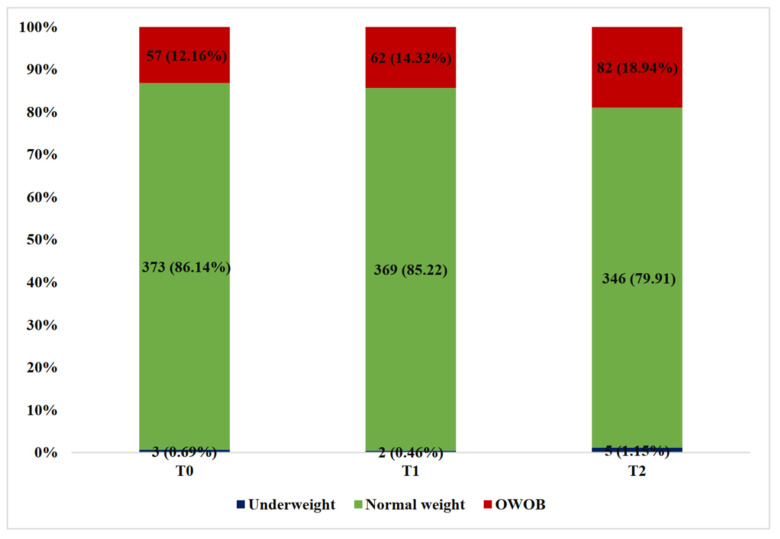
The distribution of the actual body weight classifications among the preschool children at each follow-up time point.

**Figure 3:**
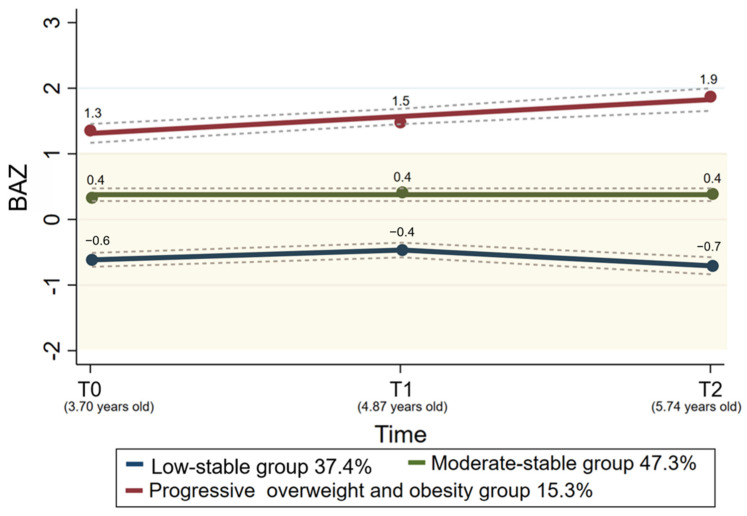
BAZ trajectories of the preschool children. Footnote: BAZ: body mass index-for-age z-score. The solid lines (blue: low-stable group; green: moderate-stable group; red: progressive overweight and obesity group) mean estimated values, and the dotted lines display the 95% CIs. The yellow zone indicates the range of the normal weight of the children (−2 ≤ BAZ ≤ 1).

The prevalence of underweight and overweight (including obesity) at T0 was 0.7% (*n* = 3) and 13.2% (*n* = 57), respectively. The rate of OWOB in the preschool children showed an upward trend in two annual follow-up visits (Figure 2). The model fitting indices indicated three BAZ trajectories among the 433 preschool children (Figure 3). (i) Group 1 (“low-stable”, *n* = 154, 37.3%) showed a stable and lower BAZ within the normal range. This group consistently maintained a BAZ ranging from −0.8 to −0.3 over two years, indicating that their weight remained consistently lower and stable. (ii) Group 2 (“moderate-stable”, *n* = 214, 47.3%) showed a stable and moderate BAZ. This group exhibited a consistent BAZ ranging from 0.2 to 0.5 over two years, indicating a stable weight within the normal range. (iii) Group 3 demonstrated a higher BAZ at baseline and maintained an upward trend over two years (“progressive overweight and obesity”, *n* = 65, 15.4%). This group was characterized by an increase in the BAZ from 1.3 at baseline, indicating that the weight was already overweight and continued to increase. In addition, the AvePP of these three trajectories were 0.92, 0.88, and 0.93, respectively. Details of the model fitting are shown in Table S1.

### 3.3. Univariate Analysis of Factors Influencing Weight Trajectories in Preschool Children

The three groups differed in demographic characteristics, parental feeding practices, and children’s appetitive traits. More children were perceived as underweight by their parents in the low-stable group than in the other two groups, and more children were perceived as overweight or obese by their parents in the progressive OWOB group (*χ^2^* = 170.69, *p* < 0.01). The score of parental pressure to eat in the progressive OWOB group was lower than in the other two groups (*F* = 3.52, *p* = 0.03). The score of children’s food responsiveness in the progressive OWOB group was higher than in the other two groups (*F* = 10.85, *p* < 0.01), and the score of children’s satiety responsiveness was lower (*F* = 10.85, *p* < 0.01) (Table 2).

### 3.4. Multinomial Logistic Regression Analysis of Factors Influencing the Weight Trajectory of Preschool Children

Compared to girls, boys were more likely to be in the progressive OWOB group than in the moderate-stable group (OR = 3.01, 95%CI: 1.44–6.25), while the likelihood of being in the low-stable group was lower (OR = 0.54, 95%CI: 0.34–0.88). High maternal BMI was associated with lower odds of being in the low-stable group than in the moderate-stable group (OR = 0.87, 95%CI: 0.80–0.96). Compared to the children perceived as normal weight by their parents, the children perceived as underweight were more likely to fall in the low-stable trajectory than in the moderate-stable group (OR = 4.57, 95%CI: 2.71–7.70). The children perceived as overweight or obese by parents were more likely to be in the progressive OWOB group rather than the moderate stable group (OR = 10.57, 95%CI: 4.89–22.86). The preschool children with a lower satiety responsiveness score at baseline were more likely to be in the progressive OWOB group than in the moderate stable group (OR = 0.86, 95%CI: 0.76–0.97) (see Table 3). In contrast to the univariate analysis, parental pressure to eat was not linked to the children’s weight trajectories according to the multinomial logistic regression analysis (*p* = 0.26).

## 4. Discussion

This two-year longitudinal study identified three distinct BAZ trajectories among the preschool children. Nearly half of the preschool children were in the moderate-stable group. Approximately one-third of the children consistently exhibited low weight and were classified as the low-stable group. The remaining children with an upward trajectory of their weight were in the progressive OWOB group. The number of trajectories reported in the previous studies of BMI trajectories in childhood ranged from three to seven, with three or four being the most common in the literature [33,34]. Several factors contribute to the smaller number of trajectories identified in this study compared to previous research. First, we focused on the weight changes in the preschoolers over two years, which is relatively shorter than other studies. Second, our follow-up interval was one year, different from the other studies. Third, amid rapid economic growth in China, there has been a steady decrease in child malnutrition, paralleled by an increasing prevalence of OWOB. This study was conducted in urban areas with a developed economy, making it challenging to capture the weight change trajectory among underweight children.

This study showed that the child’s sex, maternal BMI, parental perception of children’s weight, and child’s satiety responsiveness at baseline were associated with the weight trajectory of the preschool children across two years. Specifically, compared with the moderate-stable group, boys were more likely than girls to be in the progressive OWOB group and less likely to be in the low-stable group. A large longitudinal survey (*n* = 16,060) in China [35] also indicated that boys were more likely to have unhealthy weight trajectories, including the trajectories of “persistent obesity” and “healthy weight to overweight and obesity”. The ideal body shape differs between boys and girls in mainstream Chinese society. In China, boys with a bigger body build are often perceived as strong and healthy, while girls are commonly expected to have a thinner and slender body shape [36,37]. Therefore, caregivers usually encourage boys to consume more food and restrict food intake for girls [38]. Parents might struggle to perceive accurately or may be unconcerned about their sons’ overweight status, leading them to maintain usual feeding practices that favor a muscular physique for boys [39].

In line with previous studies, our studies demonstrated a significant association between parental weight and their children’s weight [40,41]. In addition, previous research [42,43] has shown that mothers have a stronger influence on children’s BMI than fathers, which may be due to factors such as pregnancy experiences and the maternal role as the primary caregiver for children.

The parental perception of their children in OWOB was a risk factor for progressive OWOB in the preschool children. The children perceived as overweight or obese by their parents at baseline did not effectively control their weight during follow-up but instead showed an upward trend. It was similar to two longitudinal studies in the Netherlands and Australia [44,45], which found that the parental perception of children in OWOB was associated with more weight gain in children during later follow-ups.

There are several explanations for this finding. First, within the Chinese cultural context, being overweight or obese in early childhood is often viewed as a symbol of good health. Many parents believe that weight issues in children will resolve naturally with age. Under the influence of this deeply ingrained belief, even if parents perceive that their children are overweight or obese during preschool years, they may not worry about it and maintain their original parenting practices [46,47], which may ultimately lead to a continued increase in their children’s weight. Secondly, due to weight stigma, the parents of children with OWOB may experience feeding-related stress, including concerns about their children’s health and social evaluation [48,49].

However, this concern rarely translates into healthier and more reasonable feeding practices or dietary structures within the family; instead, parents tend to adopt nonresponsive parenting—such as restricting high-calorie foods and urging children to exercise [50,51]—this type of parenting damages children’s ability to self-regulate their energy intake and expenditure. For example, children tend to consume more restricted foods or disrupt their satiety signals [52,53].

The appetitive traits directly impacted weight trajectories, where lower SR in the preschool children was associated with a progressive weight trajectory of OWOB. Overall, this study aligned with the findings of Demir and Lin [54,55], who identified FR and SR as influencing factors in childhood obesity among 1201 school-aged children. Consequently, this mechanism may increase the likelihood of unhealthy weight outcomes in children. However, this study showed that FR was not associated with the children’s weight trajectories. Indeed, parents usually determine the type and amount of food their children intake, so children’s food responsiveness is relatively limited.

Parental pressure to eat (one of the feeding practices) was found to show statistical differences among the three BAZ trajectories by the univariate analyses, but such differences were no longer significant when accounting for other variables (e.g., parental weight perception and children’s appetitive traits). We speculated that newly introduced variables (parental weight perception and children’s appetitive traits) could have a direct or strong connection to children’s weight [19,20], which interfered with the relationship between parental pressure to eat and children’s weight change. To some extent, our findings may elucidate the reason for the inconsistent results in previous research regarding the relationships between feeding practices and children’s weight—child and parental factors related to child weight were considered differently. Future studies are informed to comprehensively examine children’s and parents’ characteristics for elucidating the role of parental feeding practices in children’s weight development.

This study has several limitations. First, this study focused on weight changes in preschool children over two years, which did not cover the entire preschool period. We may be restricted to revealing the complete trajectories of weight change across the preschool years. Second, children’s weight was also influenced by physical activity, sleep, and sedentary behaviors [12], which was not examined as this was beyond the objectives of this study. Third, this study used self-reported data from the caregivers of preschool children to measure feeding practice and child appetitive traits, which may introduce reporter bias. Finally, we used convenience sampling to survey preschool children and their parents from the same urban area. Among this study population, more than half of the households belong to single-child families, where grandparents actively participate in child-rearing, and their feeding practice may also impact childhood weight changes. Additionally, the majority of the families included in this study were highly educated and earned a high annual income, which may influence the generalizability of this study.

## 5. Conclusions

Preschool children are in a critical stage of growth and development, with individual variations in weight trajectory. This study identified three distinct weight trajectories: “low-stable group”, “moderate-stable group”, and “progressive overweight and obesity group”. Parental characteristics (self-reported BMI and parental perception of children’s weight) and children’s characteristics (sex and appetitive traits) were related to the weight trajectories. Male children, those perceived as OWOB by parents, and those with low satiety responsiveness were at greater risk of following a progressive OWOB trajectory. However, this study did not find an association between feeding practices and weight trajectories in children. By identifying the intrinsic and extrinsic factors associated with weight gain in children, personalized precision health management programs can be developed and tested for the high-risk population of children in OWOB.

## Figures and Tables

**Table 1:** Demographic characteristics of the participants.

Variables	Categories	Included (*n* = 433)	Excluded (*n* = 180)	t or χ2	*p*-Value
Child characteristic					
Age (years), M (SD)		3.70 (0.29)	3.71 (0.45)	−0.31 ^a^	0.76
Sex, N (%)	Male Female	232 (53.58) 201 (46.42)	84 (46.67) 96 (53.33)	2.43	0.12
BAZ, M (SD)		0.13 (0.88)	0.17 (1.00)	−0.47	0.64
	Yes	166 (38.34)	72 (40.00)		
Presence of siblings, N (%)	No	267 (61.66)	108 (60.00)	0.15	0.70
	<6	107 (29.40)	53 (29.44)		
Breastfeeding time (months), N (%)	6~12	211 (47.78)	80 (44.44)	1.59	0.45
	>12	115 (22.82)	47 (26.11)		
Parental perception of child	Perceived underweight	119 (27.48)	50 (27.78)		
weight, N	Perceived normal weight	257 (59.35)	105 (58.33)	0.08	0.96
(%)	Perceived OWOB	57 (13.16)	25 (13.89)		
Child M	Satiety responsiveness	2.71 (0.64)	2.72 (0.62)	−0.23 ^a^	0.82
appetitive traits, (SD)	Food responsiveness	2.48 (0.56)	2.50 (0.57)	−0.37 ^a^	0.71
Family characteristic					
N	Mother	330 (76.21)	140 (77.78)		
Primary caregiver, (%)	Father	103 (23.79)	40 (22.22)	0.17	0.68
	Stem family	356 (82.22)	139 (77.22)		
Family type, N (%)	Nuclear family	68 (15.70)	40 (22.22)	5.27	0.07
	Other types	9 (2.08)	1 (0.56)		
Father’s age, M (SD)		36.54 (4.65)	36.45 (4.73)	0.20 ^a^	0.84
Mother’s age, M (SD)		34.55 (3.88)	34.51 (3.76)	0.12 ^a^	0.90
Father’s BMI, M (SD)		24.30 (3.00)	24.54 (3.06)	−0.88 ^a^	0.38
Mother’s BMI, M (SD)		21.43 (2.79)	21.38 (2.65)	0.19 ^a^	0.85
	Content-restricted feeding	3.52 (0.76)	3.43 (0.79)	1.35 ^a^	0.18
	Encouraging healthy eating	4.10 (0.65)	4.06 (0.48)	0.75 ^a^	0.45
Parental feeding practices, M	Behavior-restricted feeding	4.10 (0.59)	4.05 (0.60)	0.84 ^a^	0.40
(SD)	Food as a reward	3.33 (0.79)	3.35 (0.79)	−0.36 ^a^	0.72
	Pressure to eat	3.28 (0.78)	3.19 (0.81)	1.37 ^a^	0.17
	Monitoring	4.15 (0.75)	4.03 (0.84)	1.71 ^a^	0.09
	Senior high school or below	40 (9.24)	14 (7.78)		
Father’s education level, N (%)	College or above	393 (90.76)	157 (86.22)	0.17	0.68
Mother’s education level, N (%)	Senior high school or below college or above	41 (2.90) 391 (90.52)	15 (8.33) 157 (86.22)	0.08	0.77
Annual household income, N (%)	< RMB 300,000 > RMB 300,000	183 (42.26) 250 (57.73)	66 (36.67) 100 (55.56)	0.21	0.58

Footnote: M—mean; SD—standard deviation; N—number of cases; χ2—chi-square test; t—*t*-test; ^a^—t value.

**Table 2:** Univariate analysis of the trajectory of weight in the preschool children.

		Low-Stable Group (*n* = 154)	Moderate-Stable Group (*n* = 214)	Progressive OWOB Group (*n* = 65)	F or χ2	*p*-Value
Child characteristic						
	Male	73 (47.40)	118 (55.14)	41 (63.08)	4.93	0.09
Sex, N (%)	Female	81 (52.60)	96 (44.86)	24 (36.92)		
Age (years),	M (SD)	3.72 (0.28)	3.68 (0.30)	3.72 (0.29)	1.11 ^b^	0.33
BAZ,	M (SD)	−0.66 (0.47)	0.32 (0.56)	1.38 (0.64)	**350.72 ^b^**	**<0.01**
	Underweight	3 (1.95)	0	0	**166.24**	**<0.01**
Actual weight level, N (%)	Normal weight	151 (98.05)	197 (92.06)	25 (38.46)		
	OWOB	0	17 (7.94)	40 (61.54)		
Presence of siblings, N (%)	Yes	60 (38.96)	77 (35.98)	29 (44.62)	1.61	0.44
	No	94 (61.04)	137 (64.02)	36 (55.38)		
Breastfeeding time (months), N (%)	<6	36 (23.38)	62 (28.97)	9 (13.85)	7.50	0.11
	6~12	77 (50.00)	101 (47.20)	33 (50.77)		
	>12	41 (26.62)	51 (23.83)	23 (35.38)		
Parental perception of child weight, N (%)	Perceived underweight	82 (53.25)	37 (17.29)	0	**170.69**	**<0.01**
	Perceived normal weight	71 (46.10)	155 (72.43)	31 (47.69)		
	Perceived OWOB	1 (0.65)	22 (10.28)	34 (52.31)		
Child appetitive traits, M (SD)	Satiety responsiveness	2.85 (0.66)	2.71 (0.61)	2.43 (0.59)	**10.40**	**<0.01**
	Food responsiveness	2.33 (0.58)	2.53 (0.55)	2.69 (0.49)	**10.85 ^b^**	**<0.01**
Family characteristic						
Primary caregiver, N (%)	Mother	116 (75.33)	161 (75.23)	53 (81.54)	1.20	0.55
	Father	38 (24.67)	53 (24.77)	12 (18.46)		
Family type, N (%)	Stem family	126 (81.82)	181 (84.58)	49 (75.38)	3.74	0.44
	Nuclear family	24 (15.58)	29 (13.55)	15 (23.08)		
	Other types	4 (2.60)	4 (1.87)	1 (1.54)		
Father’s age, M (SD)		36.47 (4.63)	36.37 (4.74)	37.42 (4.36)	1.37ᵇ	0.25
						
Mother’s age, M (SD)		34.63 (4.04)	34.31 (3.87)	35.17 (3.53)	1.28 b	0.28
Father’s BMI, M (SD)		23.73 (2.86)	24.54 (3.08)	24.89 (2.92)	**4.73 ^b^**	**<0.01**
Mother’s BMI, M (SD)		20.80 (2.79)	21.72 (2.75)	21.96 (2.69)	**6.46** **^b^**	**<0.01**
	Content-restricted feeding	3.43 (0.74)	3.58 (0.77)	3.57 (0.78)	1.83 ^b^	0.16
Parental feeding practices, M (SD)	Encouraging healthy eating	4.11 (0.48)	4.08 (0.48)	4.14 (0.56)	0.48 ^b^	0.62
	Behavior- restricted feeding	4.14 (0.58)	4.07 (0.59)	4.07 (0.59)	0.68 ^b^	0.51
	Food as a reward	3.31 (0.75)	3.37 (0.81)	3.26 (0.82)	0.58 ^b^	0.56
	Pressure to eat	3.40 (0.76)	3.25 (0.78)	3.12 (0.75)	**3.52** **^b^**	**0.03**
	Monitoring	4.14 (0.67)	4.17 (0.81)	4.11 (0.74)	0.21 ^b^	0.81
Father’s education level, N (%)	Senior high school or below	16 (10.39)	19 (8.88)	5 (7.69)	0.46	0.79
	College or above	138 (89.61)	195 (91.12)	60 (92.31)		
Mother’s education level, N (%)	Senior high school or below	14 (9.09)	21 (9.86)	6 (9.23)	0.07	0.97
	College or above	140 (90.91)	192 (90.14)	59 (90.73)		
Annual household income, N (%)	< RMB 300,000	60 (38.96)	93 (43.46)	30 (46.15)	1.22	0.54
	> RMB 300,000	94 (61.04)	121 (56.54)	35 (53.85)		

Footnote: M—mean; SD—standard deviation; N—number of cases; χ2—chi-square test, F—one-way ANOVA; ^b^—F value. Estimates significant at *p* < 0.05 are in bold.

**Table 3:** Multinomial logistic analysis of the factors influencing the BAZ trajectory of the preschool children.

	Variables	B	SE	Wald χ2	*p*-Value	OR (95% CI)
Low-stable group						
Gender	Girls	Ref.				
	Boys	−0.61	0.24	6.22	0.01	**0.54 (0.34, 0.88)**
	Father’s BMI	−0.05	0.04	1.21	0.27	0.96 (0.88, 1.04)
	Mother’s BMI	−0.14	0.05	8.44	<0.01	**0.87 (0.80,0.96)**
Parental perception of child weight	Perceived normal weight	Ref.				
	Perceived underweight	1.52	0.26	32.35	<0.01	**4.57 (2.71, 7.70)**
	Perceived OWOB	−2.44	1.04	5.49	0.02	**0.09 (0.01, 0.67)**
	Pressure to eat	−0.02	0.04	0.12	0.73	0.99 (0.91, 1.07)
	Satiety responsiveness	0.01	0.04	0.05	0.82	1.01 (0.93, 1.10)
	Food responsiveness	−0.07	0.04	3.04	0.08	0.94 (0.87, 1.01)
Progressive OWOB group						
Gender	Girls	Ref.				
	Boys	1.10	0.37	8.67	<0.01	**3.01 (1.44, 6.25)**
	Father’s BMI	0.01	0.06	0.02	0.89	1.01 (0.90, 1.12)
	Mother’s BMI	0.06	0.06	1.07	0.30	1.06 (0.95, 1.19)
Parental perception of child weight	Perceived normal weight	Ref.				
	Perceived underweight	−14.31	0.30	48.77	<0.01	**0.02 (−14.89, −13.73)**
	Perceived OWOB	2.36	0.39	35.91	<0.01	**10.57 (4.89, 22.86)**
	Pressure to eat	0.06	0.06	1.28	0.26	1.07 (0.96, 1.19)
	Satiety responsiveness	−0.16	0.06	6.44	0.01	**0.86 (0.76, 0.97)**
	Food responsiveness	0.06	0.05	1.30	0.25	1.06 (0.96, 1.18)

Footnote: B—coefficient; SE—standard error; Wald χ2—Wald chi-square test; OR—odds ratio; CI—confidence interval; BMI—body mass index; OWOB—overweight and obesity. Girls and Perceived normal weight were used as the reference category. All multinomial logistic regression models used the Moderate-stable group as the reference group. Estimates significant at *p* < 0.05 are in bold.

## Data Availability

Data will be made available upon request.

## Supplementary Materials

The following supporting information can be downloaded at https://www.mdpi.com/article/10.3390/nu16213746/s1. Table S1: Fit indices in group-based trajectory model (GBTM).

## Abbreviations

BAZ: body mass index-for-age z-score; OR: odds ratio; CI: confidence interval; BMI: body mass index; OWOB: overweight and obesity; FR: food responsiveness; SR: satiety responsiveness; GBTM: group-based trajectory modeling; CPCFBS: Chinese Preschooler’s Caregivers’ Feeding Behavior Scale; C-CFQ: Chinese version of the Child Feeding Questionnaire; CPEBQ: Chinese Preschoolers’ Eating Behavior Questionnaire; EM: expectation–maximization algorithm; SD: standard deviation; BIC: Bayesian information criteria; AvePP: average posterior probability.
