# Supplementary material for: Parental Feeding Practices, Weight Perception, and Children’s Appetitive Traits Are Associated with Weight Trajectories in Preschoolers: A Longitudinal Study in China
Source: Nutrients. 2024 Oct 31;16(21):3746. doi: 10.3390/nu16213746 (PMC11547943; doi:10.3390/nu16213746)
Supplement: Supplementary file 1 [file nutrients-16-03746-s001.zip › nutrients-3259662-supplementary.pdf]

Table S1 Fit indices in group-based trajectory model (GBTM)

Number of classes	Trajectory shape (0 = zero order, 1=linear, 2=quadratic, 3=cube)	BIC (N=433)	AIC (N=433)	Entropy	Proportion of subgroup sample size (%)
	0 0	-1555.46	-1547.32	0.775	64.8/35.2
	0 1	-1552.57	-1542.39	0.782	65.6/34.4
	0 2	-1555.23	-1543.02	0.783	65.8/34.2
2	1 1	-1555.38	-1543.17	0.782	65.5/34.5
	1 2	-1558.04	-1543.80	0.783	65.7/34.3
	2 2	-1556.25	-1539.96	0.785	65.5/34.5
	3 3	-1562.32	-1541.96	0.785	65.5/34.5
	0 0 0	-1476.91	-1464.69	0.783	37.2/47.3/15.5
	0 1 0	-1479.10	-1464.85	0.783	37.0/47.6/15.4
	0 1 1	-1470.76	-1454.48	0.792	37.3/47.5/15.2
	0 1 2	-1472.44	-1454.12	0.793	37.3/47.5/15.2
	0 2 0	-1481.07	-1464.79	0.784	36.2/48.0/15.7
	0 2 1	-1472.78	-1454.46	0.792	36.5/48.0/15.5
	0 2 2	-1474.33	-1453.98	0.793	36.5/48.0/15.5
	1 0 1	-1470.28	-1453.99	0.792	36.6/47.9/15.5
	1 1 0	-1480.94	-1464.66	0.784	36.0/48.3/15.7
**3**	1 1 1	-1472.63	-1454.37	0.792	36.3/48.2/15.5
	1 1 2	-1474.36	-1454.01	0.794	36.3/48.2/15.5
	1 2 1	-1474.65	-1454.29	0.794	35.6/48.7/15.7
	1 2 2	-1476.19	-1453.80	0.795	35.6/48.7/15.7
	**2 0 1**	**-1467.41**	**-1449.09**	**0.794**	**37.3/47.3/15.4**
	2 1 1	-1469.78	-1449.43	0.794	37.0/47.7/15.3
	2 2 1	-1472.11	-1449.72	0.795	36.4/48.1/15.5
	2 2 2	-1473.66	-1449.24	0.796	36.3/48.1/15.6
	2 2 3	-1476.70	-1450.24	0.796	36.3/48.1/15.6
	2 3 3	-1479.73	-1451.24	0.796	36.3/48.1/15.6
	3 3 3	-1482.77	-1452.24	0.796	36.3/48.1/15.6
4	0 0 0 0	-1449.07	-1432.78	0.823	33.7/46.8/18.2/1.3

Footnote: this study selected the three trajectories with polynomials 2,0,1.
